# Vivisectors and Their Work

**Published:** 1889-04-06

**Authors:** 


### Within the Wards.

### VIVISECTORS AND THEIR WORK.

#### Point of View.

To bind a living, sentient animal firmly to a table, and to take sharp instruments and cut as freely and deeply into any part of its body as if it were dead or without feeling, is a practice which, at the first blush, revolts and horrifies the highly sensitive mind of the nineteenth century. It should be considered from the point of view of the animal as well as from that of man. Not, however, from the animal's point of view mainly, much less exclusively. For if the animals were allowed to decide what they would and would not do and endure for man, we should not only have no more beef to eat, no more fish, and no more game, but we should have no more horses to ride upon or to draw our carts and carriages, no more cows to milk, no more sheep to shear for clothing, and no more menageries and Zoological Gardens to delight the children and instruct the grown-up people. Nay, more, if the view of deciding all questions affecting animals exclusively from the animals' standpoint were logically carried out, the wolves would eat the cattle of Northern villages without complaint, and would even eat the villagers themselves in case of need or preference; the tigers of India and the lions of Africa would depose man from his supremacy and reign in his stead; and even bugs and fleas would put in a claim for protection, and increase and multiply and be happy at the expense of man without let or hindrance All this, and much more, would be the natural and inevitable consequence of deciding all questions affecting the lower animals from the animals' standpoint alone. Clearly, therefore, that cannot be done.

#### Is the Practice Justifiable?

Does it follow from this that vivisection is justifiable? By no means! But it does follow that the question of its justifiableness is worthy of the gravest consideration, and, moreover, that it can only be fittingly considered by those who thoroughly inform themselves on the subject. Two competent vivisectors have recently published details of a series of experiments on living animals, undertaken with the view of determining whether or not a certain method which has occasionally been suggested and employed for the cure of apoplexy is rational and likely to be successful. Now, all the world knows that the ordinary treatment of apoplexy is strictly of the kind which may be termed "waiting upon Providence." In apoplexy a blood-vessel of greater or less size has been ruptured. The blood begins to flow out of the ruptured vessel into the brain in that form of apoplexy which is known as "cerebral." If the blood flow for a sufficient length of time and in adequate quantities, no mortal power can save the patient's life. And yet, if one could but put a pad on the ruptured vessel immediately after the rupture, or could do what is done by many a policeman and young lady who has had ambulance lessons, and compress the main artery that leads to and supplies the one that is bleeding the patient to death in the brain, the dangerous symptoms would cease almost immediately. That, at any rate, has hitherto been the assumption, and it was with the view of testing the accuracy of that assumption that the two vivisectors, Mr. Victor Horsley, F.R.S., and Mr. Walter Spencer, M.S., initiated and carried out their series of experiments.

#### Monkeys Selected.

The animals selected for experiment were Macaque monkeys (*Macacus Sinicus* and *Macacus Rhesus*). Those were chosen because of the general similarity of brain which exists between them and man, but especially because of the practical identity of the blood circulation in the two. The arrangement of the blood-vessels in several species of mammalian animals somewhat resembles that of man—for example, that of the horse, the dog, the rabbit, and certain monkeys. But in none of these is the similarity nearly so marked as in the macaques. It may, therefore, be considered that an experiment on such a monkey, in so far as it relates to the blood circulation in the brain, is practically an experiment on a human subject.

#### Anæsthetics Used.

In carrying out their operations, the following methods of procedure were adopted by the vivisectors; and as these methods are a fair sample of those generally employed by British physiologists, readers who desire to gain some actual knowledge of the subject will do well to note the proceedings step by step. The first thing they did was to put the monkey operated upon completely under the influence of an anæsthetic. It could not in that case have either knowledge or feeling of what was being done to it. If it be said that that would render the result of the experiment doubtful, the answer is "No," because the facts to be ascertained in this case referred principally, indeed almost exclusively, to the blood circulation, and the blood circulation continues in full force whilst animals are anæsthetised. A portion of the skullcap was then gently and carefully removed, and that part of the brain laid bare which it was desired more particularly to examine. The point to be decided by the experiments was whether and to what extent the circulation could be modified in the branch arteries of that part of the brain by compressing the main trunk artery in the neck. The main trunk artery in the neck is the *carotid*, and it is possible, by pressing it firmly against the hard bones of the vertebral column, to diminish or entirely check its onward flow.

*(To be continued.)*

